# A Year of Progress and Financial Prosperity

**Published:** 1921-01-29

**Authors:** 


### LEICESTER ROYAL INFIRMARY.

### A Year of Progress and Financial Prosperity.

THE Report submitted last week to the meeting of the Governors of the Leicester Royal Infirmary (presided over by Mr. T. Fielding Johnson, jun., J.P.) outlined a large post-war scheme of development undertaken.

### EXPENDITURE ON SITE, BUILDINGS, AND EQUIPMENT.

At a cost of £20,000 additional bedrooms are being provided in the "Sir Edward Wood" Nurses' Home for sixty-six new members of the nursing staff. Pending the completion of these rooms, a Home formerly used as a nursing institution, near by the infirmary, has been acquired as a temporary Home for the infirmary nurses at a cost of several hundred pounds.

The new orthopædic department, pathological laboratories, mortuary, and viewing chapel, which were all opened in July, cost £20,000.

Excavations have been commenced on a new south-west wing with a view to the foundations of the structure being completed early this year. This wing will contain ninety-three beds and will be constructed with iron and glass balconies on all three floors along the entire length and width. The architect's estimate of the cost is £45,000. This sum has been raised as to £22,500 from the executors of the late Mr. T. G. Langham (Langham Memorial Fund), £10,000 from the British Red Cross Society, and £10,000 from the Leicester and Leicestershire Prisoners of War Parcels Fund. The cost of furnishing will be £15,000.

A new venereal diseases department has been approved by the Ministry of Health, and it is expected this will shortly be commenced. The capital cost will be provided by the institution in return for a rent of 7 per cent. on the entire cost of building and furnishing, estimated at £12,000. Nine beds for women and children in separate cubicles are provided for in-patients for the same department at a cost of £400. An additional ward of twelve beds for female surgical cases has been equipped at a cost of £500.

Additions have been made to the site area by the purchase of properties at a cost of £2,500.

### THE WORK OF THE YEAR AND ITS COST.

The work of the year in all departments shows considerable increase. Admissions totalled 4,580, practically 45,000 out-patients received treatment, 67,000 accident attendances were recorded, 4,619 cases passed through the x-ray department, and operations numbered 5,848. The pathological department made 8,416 examinations and reports, compared with 5,844, while 19,885 treatments were given in the new orthopædic department.

The year's expenditure reached the high figure of £64,130, while the income from all sources brought into account was approximately £61,000, so that the year closed with a deficit of £3,200. Annual subscriptions produced £9,420, an increase of approximately £500 on the previous year. This is a proof of the stability of the contribution scheme founded by Mr. Alfred Corah, the late treasurer.

Donations amounted to £10,620, and, allowing for investments, £8,436 was available for income. Dividends at £4,600 showed a considerable increase. Contributions on Hospital Sunday, etc., showed an increase of £340, while the Saturday Hospital Society, in consequence of the doubling of its contributions, returned the sum of £19,000, as compared with £14,375. Legacies amounted to £8,190, £2,700 being for bed endowments. Property purchased cost £2,500, and the balance, £3,000, was carried to the credit of income for the year. On the Children's Hospital account £3,200 was available as income for the year, after investing £867 for particular contributions. Receipts amounting to £6,000 were included as income for the year, which in 1919 had been capitalised, but against this the total amount invested for the year amounted to upwards of £9,000.

The Report emphasises the need for co-ordination of services between the local hospitals. Many patients were received from towns having their own hospitals, and in order to take in cases from distant areas, lesser cases of accident from districts near to hand were dealt with as out-patients, though they might much more effectively have been treated as in-patients. The latter cases were generally contributors to the institution, while the former might not be. The need for reciprocity between the institutions and an arrangement for an interchange of patients is therefore indicated, and it is suggested that the plan proposed by Lord Dawson and Colonel Bond, Chairman and Deputy-Chairman of the Ministry of Health Consultative Committee on Medical Services, to make use of available accommodation in Poor-law institutions might be considered.

### £150,000 AS AN ANNIVERSARY CELEBRATION.

The proposal to celebrate the 150th anniversary this year coincides with a period of commercial depression, but it is hoped to secure modified help from many sources rather than large help from limited sources. Efforts will be made to augment the annual income as well as to provide a capital sum for the sixty-six bedrooms now being erected for nurses, and the new wing of 100 beds will be a large charge upon the annual income. The building scheme involves £20,000 for the additions to the Nurses' Home, £15,000 for furnishing the new wing, and £5,000 to complete the pathological department. £100,000 to £150,000 should therefore be aimed at.

